# Establishing National Multisectoral Coordination and collaboration mechanisms to prevent, detect, and respond to public health threats in Guinea, Liberia, and Sierra Leone 2016–2018

**DOI:** 10.1186/s42522-019-0004-z

**Published:** 2019-11-27

**Authors:** Serge Agbo, Lionel Gbaguidi, Chethana Biliyar, Seydou Sylla, Mukeh Fahnbulleh, John Dogba, Sakoba Keita, Sarian Kamara, Amara Jambai, Albert Harris, Tolbert Nyenswah, Mane Seni, Sow Bhoye, Sambe Duale, Andrew Kitua

**Affiliations:** 1 USAID/EPT-2 Preparedness and Response Project (P&R Project), Abidjan, Ivory Coast; 2 USAID/EPT-2 Preparedness and Response Project (P&R Project), Conakry, Guinea; 3 USAID/EPT-2 Preparedness and Response Project (P&R Project), Freetown, Sierra Leone; 4 USAID/EPT-2 Preparedness and Response Project (P&R Project), Monrovia, Liberia; 5 Ministry of Health (MOH), Conakry, Guinea; 6 Ministry of Health (MOH), Freetown, Sierra Leone; 7 Ministry of Health (MOH), Monrovia, Liberia; 8 Ministry of Agriculture –Livestock, Conakry, Guinea; 9 Ministry of Environment, water and Forestry, Conakry, Guinea; 10 USAID/EPT-2 Preparedness and Response Project (P&R Project) –DAI Global, Bethesda, Maryland USA; 11 USAID/EPT-2 Preparedness and Response Project (P&R Project) –Africa, Kihonda, White House Plot 980, /81 Morogoro, Tanzania

**Keywords:** One health, National one Health Platform, Platform, Multisectoral coordination and collaboration, Multisectoral coordination mechanism

## Abstract

**Background:**

The governments of Guinea, Liberia, and Sierra Leone have acknowledged that weak health systems and poor coordination of efforts hampered effectiveness of the 2014–2016 Ebola outbreak response. The bitter experience of the Ebola outbreak response served as an important catalyst for increased efforts to comply with World Health Organization (WHO) International Health Regulations (IHR 2005), Performance of Veterinary Services (PVS) Pathway capacities, and Global Health Security Agenda (GHSA) goals. In November 2016, an interministerial meeting held in Dakar, Senegal, resulted in formalized commitments from the three nations to strengthen resilience to health threats by establishing a Regional Strategic Roadmap to institutionalize the One Health approach. Since then, each country has made significant progress towards establishing National One Health Platforms to coordinate health security interventions, in collaboration with international partners. This paper outlines the methodology and results of these efforts for the period June 2016–January 2019, with a specific focus on activities supported by the US Agency for International Development (USAID)-funded Preparedness & Response (P&R) project.

**Objectives:**

In support of the West African Health Organization’s November 2016 Regional Strategic Roadmap for institutionalization of the One Health approach, the Preparedness & Response (P&R) project worked in coordination with national partners in Guinea, Liberia, and Sierra Leone to establish multisectoral, One Health coordinating mechanisms.

**Methodology:**

The global USAID-funded P&R project was launched in 2014 to support the achievement of this objective, and began coordinating with partners in Guinea, Liberia, and Sierra Leone in 2016 to tailor its multi-step conceptual framework to fit the priorities and operating constraints of national stakeholders. Organized in phases of Collaboration (building key relationships), Formalization (defining and establishing a coordination structure), and Implementation (using newfound coordination to produce better health security outcomes), the framework features steps such as One Health sensitizations for multisectoral national stakeholders, development of One Health platform terms of reference and other operating guidelines, and application of these tools to coordination of technical assistance during outbreaks.

**Results:**

In Guinea, Liberia, and Sierra Leone, in less than 3 yrs there has been a marked improvement in cross-sectoral coordination on health security actions. All three countries have passed legislation establishing permanent multisectoral coordination mechanisms referred to in this document as National One Health Platforms, or simply Platforms; instituted an annual mechanism for assessing capacity and performance of these platforms to lead health security actions; and have undertaken key steps towards developing and updating National Preparedness & Response Plans which truly reflect the multisectoral nature of emerging disease threats. However, multisectoral coordination is a work in progress: government stakeholders and their international partners continue to work together to further strengthen national ownership and investment in the newly established Platforms.

**Conclusion and next steps:**

Newly established Platforms in Guinea, Liberia, and Sierra Leone offer a long-term structure for coordinating health security actions. However, given the short period of time since their formalization, they depend on continued national, regional, and international resources to build from recent progress and further improve capacity and performance. Regional programs such as the World Bank Regional Disease Surveillance Systems Enhancement (REDISSE) project are of critical importance in keeping the momentum going. The highlighted progress and outputs to date provide reasons and motivation for continued, longer-term investment in the Platforms.

## Background

The Economic Community of West African States (ECOWAS) faces ongoing threats from zoonosis and antimicrobial resistance and suffered serious socioeconomic consequences during the 2014–2016 Ebola virus disease outbreak. Weak health systems and poor coordination of efforts were among the major factors that hampered the effectiveness of the initial response to the Ebola outbreak in Guinea, Liberia and Sierra Leone [1–4]. This was further compounded by mistrust among indigenous communities of government officials, international organizations, and foreign agencies; fear of some of the prevention and response interventions; and poor logistical support during the epidemic [3].

The outbreak, among other health threats, prompted the establishment of the Global Health Security Agenda (GHSA), a partnership of over 64 nations, international organizations, and non-governmental stakeholders engaged in building countries’ capacity to address infectious disease threats and invest in global health security [5]. Recognizing the need for cross-sectoral collaboration, stakeholders at the national level began to call for stronger political commitments to building long-term resilience against public health threats [5, 6].

The West African Health Organization (WAHO) responded by coordinating with other regional and international bodies to develop a draft Regional Strategic Roadmap for institutionalizing the One Health approach to strengthen prevention, detection, and response to infectious diseases in ECOWAS member states. The Roadmap was validated through a series of technical and ministerial meetings involving 15 ECOWAS countries (Dakar, Senegal in November 2016) followed by an inter-ministerial meeting in Abuja, Nigeria in 2017 [7–11]. Member states signed policy and financial commitments to strengthen national preparedness and response capacities against public health threats through institutionalization of the One Health approach. They also called on international partners’ support in carrying out the Joint External Evaluation (JEE) of core capacities to meet the requirements of the World Health Organization (WHO) International Health Regulation (IHR 2005) [12] and in assessing the state of veterinary services using the Office International des Epizooties (OIE) tool for the Evaluation of Performance of Veterinary Services (OIE PVS tool) [13].

The resolve of the ECOWAS countries to strengthen multisectoral preparedness and response capacities to prevent, detect, and respond to emerging public health threats attracted support from regional donor initiatives funded by agencies like the US Agency for International Development (USAID) and World Bank, respectively the Emerging Pandemic Threats 2 (EPT2) suite of projects and the Regional Disease Surveillance Systems Enhancement (REDISSE) project [14, 15].

The Preparedness & Response (P&R) project was a component of the USAID/EPT2 program specifically focused on supporting countries to build strong Multisectoral Coordinating Mechanisms (MCM), herein referred to as Platforms. Through the lens of the P&R project, this paper outlines the process and highlights progress towards One Health institutionalization in Guinea, Liberia, and Sierra Leone from June 2016 to January 2019, including new legislations, structures, terms of references, and strategic plans.

## Objectives

In support of the West African Health Organization’s November 2016 Regional Strategic Roadmap for institutionalization of the One Health approach, the Preparedness & Response (P&R) project worked in coordination with national partners in Guinea, Liberia, and Sierra Leone to establish multisectoral coordinating mechanisms.

## Methodology

The P&R project began its support to Guinea, Liberia, and Sierra Leone in 2016, having implemented similar activities in the East Africa and Southeast Asia regions. The project’s global conceptual framework, specifying three thematic areas of intervention (Collaborate, Formalize, and Implement), was adapted to fit stakeholder needs in West Africa. Table 1 below summarizes key actions under each thematic area and includes additional actions which contribute to the enabling environment for institutionalization of the One Health approach.

**Table 1:** Main Elements of the Conceptual Framework - Establishing National One Health Platforms

Collaborate	Formalize	Implement	
Map One Health stakeholders	Define platform roles and responsibilities	Review, update, or develop national One Health Strategic Plan	Develop or review guidelines, SOPs, and protocols
Organize One Health sensitization	Develop organizational structure	Conduct preparedness & response simulations	Review, update, or develop National Preparedness & Response Plans
Facilitate multi-stakeholder coordination meetings	Launch platform	Facilitate after-action reviews (AARs)	Provide technical assistance for outbreak response
	Strengthen platform operations	Conduct prioritization of zoonotic diseases	Strengthen health security interventions
*Fostering Enabling Environment:*	*Support One Health policy advocacy; Engage private sector stakeholders; Support gender integration; mobilize technical and financial resources*		

The two thematic areas “collaborate” and “formalize” address the establishment of the Platform in terms of its structure and functions, while the thematic area “implement” addresses core One Health coordination activities to be carried out by the Platform [16].

Best practices from the P&R project’s collective experience within and outside West Africa have been compiled and formalized in a series of “toolkits” [16–18]. Although specific approaches varied by country due to contextual considerations, the toolkits served to standardize the approaches.

### Collaborate

In Guinea, Liberia, and Sierra Leone, national technical experts and facilitators organized One Health stakeholder mapping exercises, sensitizations, and multisectoral coordination meetings in consultation with national partners. Stakeholder mapping was conducted as the first activity in order to identify key One Health championing institutions, supporters, and other resources for coordination and collaboration. P&R then organized sensitization sessions to lay the foundation for future One Health activities led by these critical partners. In Liberia, due to limited resources, the stakeholder mapping sessions were paired with One Health sensitization sessions, ensuring participation despite busy schedules and competing priorities.

The establishment of quarterly multisectoral coordination meetings further improved trust and cross-sectoral coordination. Led by an individual appointed by majority consensus (often a designated “platform chair”), the meetings allowed for joint planning, resource management, and troubleshooting of challenges, as well as general progress updates on implementation of One Health activities.

### Formalize

Once national stakeholders expressed more comfort with the principles of multisectoral coordination, they were able to engage in the process of formalizing the structure and composition of the MCM (Platform). The Platform was defined broadly as a structure that serves as a multisectoral coordination mechanism designed to address health threats including emerging disease threats, endemic diseases, AMR, disasters and environmental toxins among others [19].

**Fig. 1:**
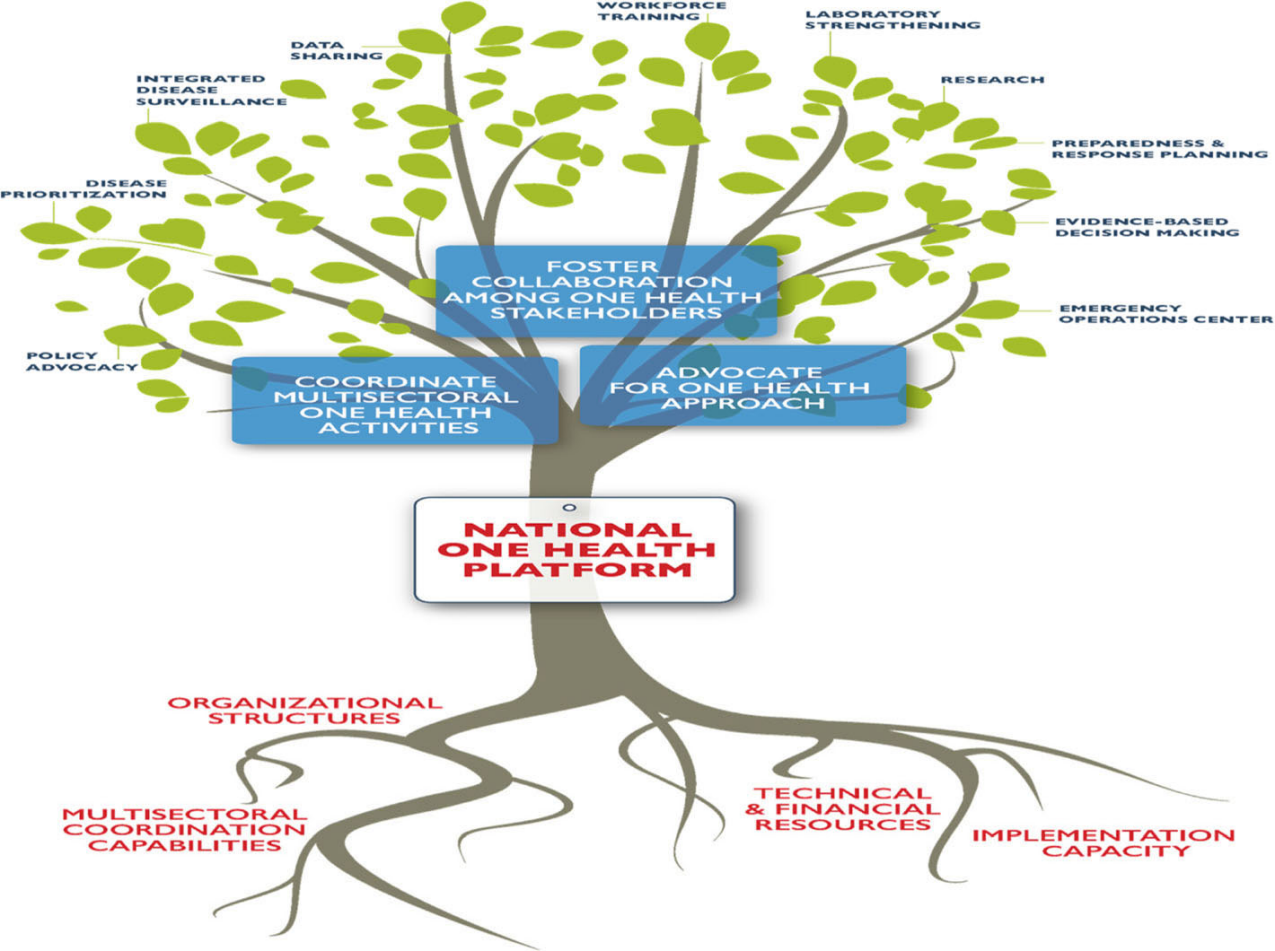
The Structure and Responsibilities of the National One Health Platform

Figure 1 below summarizes the structure and functions of a National One Health Platform in pictorial form [16]. The roots represent the partners and resources that are foundational to the Platform (stem of the tree), while the branches represent the key functions of the platform and the leaves are the activities to be implemented through joint multisectoral collaboration.

In coordination with One Health partners, consultations were carried out with key stakeholders followed by a series of pre-validation and validation workshops to develop additional documents formalizing the Platform such as Terms of Reference, and Organizational Charts noting key individuals to staff or be seconded to the Platform from sector-specific ministries. This lengthy process, best practices for which are summarized in the toolkits, included desk research and interviews of government personnel from each of the three key sectoral ministries.

### Implement

With the formal Platform structure in place, a new mechanism had been established to execute One Health activities including strategic plans, zoonotic disease prioritization exercises, and National Preparedness and Response Plans targeting specific diseases through a One Health lens.

Activity implementation drew on complementary national and international resources. For example, National One Health Strategic Plans were informed by global best practices from P&R’s strategic plan toolkit but developed by country-level technical experts applying participatory approaches to collect feedback from national One Health stakeholders [16, 17]. Main collaborating partners in this exercise included national ministry representatives from the human, animal, and environmental health sectors, academic institutions, and international partners such as WHO, USAID, and the Food and Agriculture Organization of the United Nations (FAO). A similar blend of country-level and international partners collaborated to carry out the Zoonotic Disease Prioritization exercise under the leadership of the Centers for Disease Control and Prevention (CDC) [18].

### Fostering an enabling environment

The long-term performance of newly established Platforms is dependent on sustained policy advocacy, inclusion of key stakeholders, coordination and collaboration among key sectors and stakeholders, and mobilization of technical and financial resources among other factors. Guinea, Sierra Leone, and Liberia continue to strengthen their One Health enabling environment by engaging multisectoral national and international stakeholders through the Platforms and developing domestic annual action plans that include a financial commitment from national governments.

### Monitoring National one Health Platform Capacity and performance

To monitor the capacity and performance of National One Health Platforms, the P&R project piloted the annual One Health Assessment for Planning and Performance (OH-APP) process and tool [16, 17], which takes place over three phases:

#### Phase 1

During Phase 1, a facilitator is selected from a pool of national One Health “champions” to consult with national One Health stakeholders and compile relevant policy documents, strategies and plans.

#### Phase 2

**Fig. 2:**
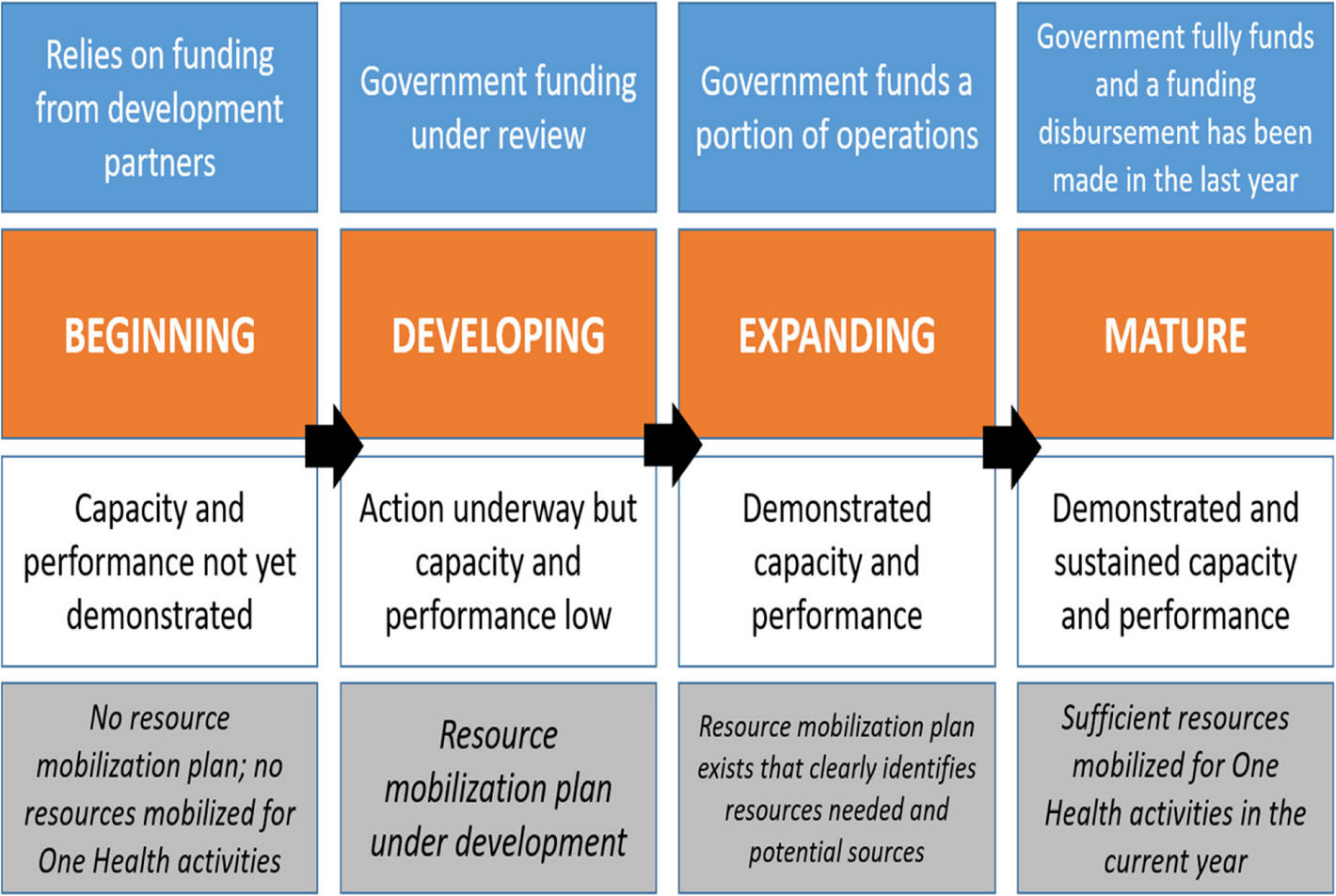
The National One Health Platform maturity model for capacity and performance

Phase 2 involves a workshop facilitated by the champion designated in Phase 1. Attendees representing national and international One Health collaborating institutions individually assess platform organizational capacity and performance, and then participate in a plenary session where all workshop participants agree by consensus on the scores for organizational capacity and performance. Scores translate to a platform “maturity level” ranging from **Beginning** to **Mature** which provides a broad picture of progress achieved to date, as seen below in Fig. 2.

#### Phase 3

Following the workshop, the final OH-APP phase is focused on producing an annual workplan for the platform informed by assessment results and overall platform maturity. A smaller team of key platform members representing the secretariat and technical working groups draft the workplan, which is presented and discussed at the next platform coordination meeting for approval.

The OH-APP tool complements the WHO IHR Joint External Evaluation (JEE) by providing a means of measuring the Platform’s growing ability to carry out multisectoral actions prioritized under the JEE.

## Results

### Collaborate

In total, 31 key multisectoral meetings were conducted from 2016 to 2018 to sensitize and build consensus with stakeholders and senior government leadership in Guinea (13), Liberia (6), and Sierra Leone (12). In addition, every country conducted regular quarterly Platform coordination meetings for planning and monitoring progress. These numerous meetings with groups and individual stakeholders resulted in increased knowledge of One Health and a consensus on the values of using the One Health approach to strengthen national preparedness and response capacity. For example, in Sierra Leone, multisectoral meetings of stakeholders from the human, animal, and environmental health sectors have resulted in animal health data being included in weekly emerging disease statistics disseminated by the National Public Health Agency.

### Formalize

Guinea, Liberia, and Sierra Leone adopted slightly different National One Health Platform structures which reflect country-specific institutional relationships and other contextual considerations. In Guinea, the One Health Platform is anchored at the inter-ministerial level and chaired by the Ministry of Health. In Liberia, the Platform is hosted by the National Public Health Institute of Liberia but sits under the Office of the Vice President from a policy perspective. In Sierra Leone, the Platform is anchored in the Ministry of Health and Sanitation, but co-chaired by the three key stakeholder entities of Ministry of Agriculture and Forestry, the Environmental Protection Agency, and Ministry of Health and Sanitation.

Table 2 below summarizes key attributes of Platforms in Guinea, Liberia, and Sierra Leone:

**Table 2:** Institutional anchorage, structure and other attributes of the National One Health Platforms of Guinea, Liberia and Sierra Leone

	Guinea	Liberia	Sierra Leone
Platform “Anchor” Institution and chairs	Anchored at interministerial level, chaired by Ministry of Health	Anchored under Office of the Vice President with secretariat managed by National Public Health Institute of Liberia	Anchored at Ministry of Health, chaired by Ministry of Agriculture and Forestry, Environmental Protection Agency, and Ministry of Health and Sanitation
Number of Platform secretariat permanent members	4	8	4
Number of Platform secretariat non-permanent members	5	2	6
Total number of Platform secretariat members	9	8	10
Permanent Platform secretariat members	• Permanent Secretary • M&E Officer • Communications Officer • Administrative Assistant	• One Health Coordinator • M&E Officer • Communications Officer • Administrative Assistant • Focal points from the animal, wildlife, human, and environmental health sectors (4 individuals)	• Permanent Secretary • Monitoring and Evaluation (M&E) Officer • Communications Officer • Administrative Assistant
Non-Permanent Platform secretariat members	• One focal point from the animal health sector • One focal point from the human health sector • One focal point from the environment sector • Representatives from international partners such as WHO and FAO	• Representatives from international organizations	• Two focal points from the animal health sector • Two focal points from the human health sector • Two focal points from the environment sector • Representatives from international partners such as WHO and FAO
Number of permanent Technical Working Groups (TWGs)	4	5	4
Total number of TWGs	4 permanent + additional temporary structures	5 permanent + additional temporary structures	4 permanent + additional temporary structures
Permanent TWGs	Surveillance Laboratory Immunization • Point of Entry	• Surveillance • Laboratory • Preparedness and Response • Antimicrobial Resistance (AMR) • Human Resource / workforce	• Surveillance • Laboratory • Immunization • Point of Entry
Expected Ad Hoc TWGs	• AMR	• Not defined	• AMR

Though complex, definition of Platform roles and responsibilities, along with its structure and composition, marks only the first step in formalizing the MCM. With stakeholder buy-in in place, each country next began the official political process to pass policy legislation establishing the Platform.

In Liberia, this document took the shape of a Declaration of Commitment signed between all implicated ministries, including the Ministry of Health, Ministry of Agriculture, Environmental Protection Agency, and Forestry Development Authority as well as others. In Guinea, the Platform was formalized via Government Joint Order, and in Sierra Leone, a Memorandum of Understanding. Though the number of participating ministries varied from country to country, a common element is that each document required representatives from key One Health sectors to commit to long-term collaboration using the Platform. Table 3 presents the key milestones achieved in Guinea, Liberia and Sierra Leone leading to the official launch of their national platforms.

**Table 3:** Key milestones formalizing National One Health Platforms in Guinea, Liberia, and Sierra Leone

Formalization Milestone	Guinea	Liberia	Sierra Leone
One Health stakeholder mapping, sensitizations, and formative multisectoral stakeholder meetings	October 2016 – May 2018	October 2016 – September 2018	April 2016 – September 2018
Platform roles and responsibilities defined; governance manual developed*	February 2017 *Governance Manual:* May 2018	October 2017–September 2018 *Governance Manual:* February 2018	September – November 2016 *Governance Manual:* October 2018
Platform organizational structure defined	September – November 2016	October to November 2016	September – November 2016
Platform focal points designated	October – November 2016	*In process*	May 2017
Legal framework established	July 31, 2017 *Key features:* Interministerial Government Joint Order signed by the Ministry of Health, Ministry of Environment, Water, and Forests, Ministry of Livestock and Animal Production, and Secretary General	June 28, 2017 *Key features:* Declaration of Commitment between Ministry of Health, Ministry of Agriculture, Ministry of Internal Affairs, Ministry of Foreign Affairs, Ministry of Commerce and Industry, National Disaster Management Agency, Forestry Development Authority, Environmental Protection Agency, the National Public Health Institute, and Office of the Vice President.	June 15, 2017 *Key features:* Memorandum of Understanding (MoU) signed by Ministry of Health, Ministry of Agriculture and Forestry, Environmental Protection Agency, and additional line ministries
Platform launched	April 2017	June 2017	June 2017

*The governance manuals are more robust documents outlining procedures for platform members to follow when carrying out their duties. Though this document is part of the “formalize” component, it may be developed only after platform launch

**Fig. 3:**
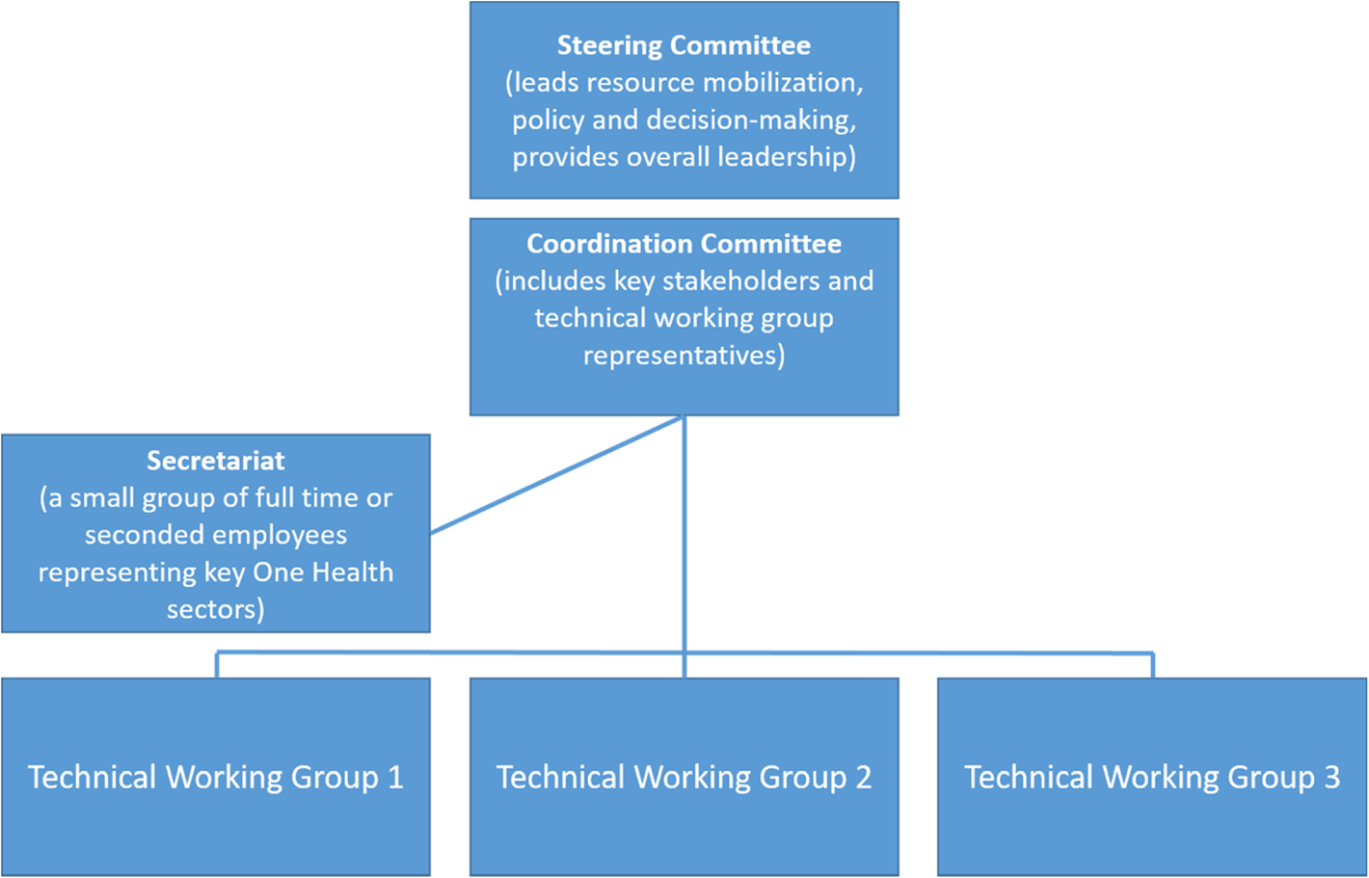
Main features of the platform organizational structure

Each of the three countries has achieved a consensus on its platform’s structure, policies, and legal framework, and has officially launched its platform. Each country has also validated a formal governance manual to clarify the roles and responsibilities of administrative and technical stakeholders engaged in platform activities, enhancing transparency, efficiency, and sustainability of platform operations. Figure 3 below highlights some common elements to each platform’s organizational structure:

### Implement

Establishment of the Platforms in Guinea, Liberia and Sierra Leone has greatly facilitated the development of national resources such as cross-cutting National One Health Strategic Plans, annual action plans, and disease-specific National Preparedness & Response Plans. Liberia’s National Action Plan on Prevention and Containment of Antimicrobial Resistance was validated efficiently by representatives from all sectors in the year 2018, in conjunction with the National One Health Strategic Plan. Table 4 summarizes key outputs of implementation as of January 2019:

**Table 4:** Key One Health Implementation Outputs by January 2019 in Guinea, Liberia, and Sierra Leone

Implementation Output	Guinea	Liberia	Sierra Leone
Review or update to cross-cutting National One Health Strategic Plan	June – September 2018	March – September 2018	April – September 2018
Zoonotic Disease Prioritization Exercise	Not conducted	not conducted	November 2017
National One Health capacity and performance assessment training	13–14 Feb 2018	October 10–11, 2018	3–4 May 2018
Review or update to disease-specific National Preparedness and Response Plans*	Conducted outside this project support	December 2016: Ebola Preparedness and Response Plan review* March – September 2018: Validation of National Action Plan (NAP) on Prevention and Containment of Antimicrobial Resistance (AMR), including disease epidemiology surveillance, laboratory surveillance, and AMR surveillance	Conducted outside this project support

*Note that this activity was not managed by USAID/P&R, but represents an important contribution of technical resources from Liberia National One Health Platform members

### Fostering an enabling environment

**Fig. 4:**
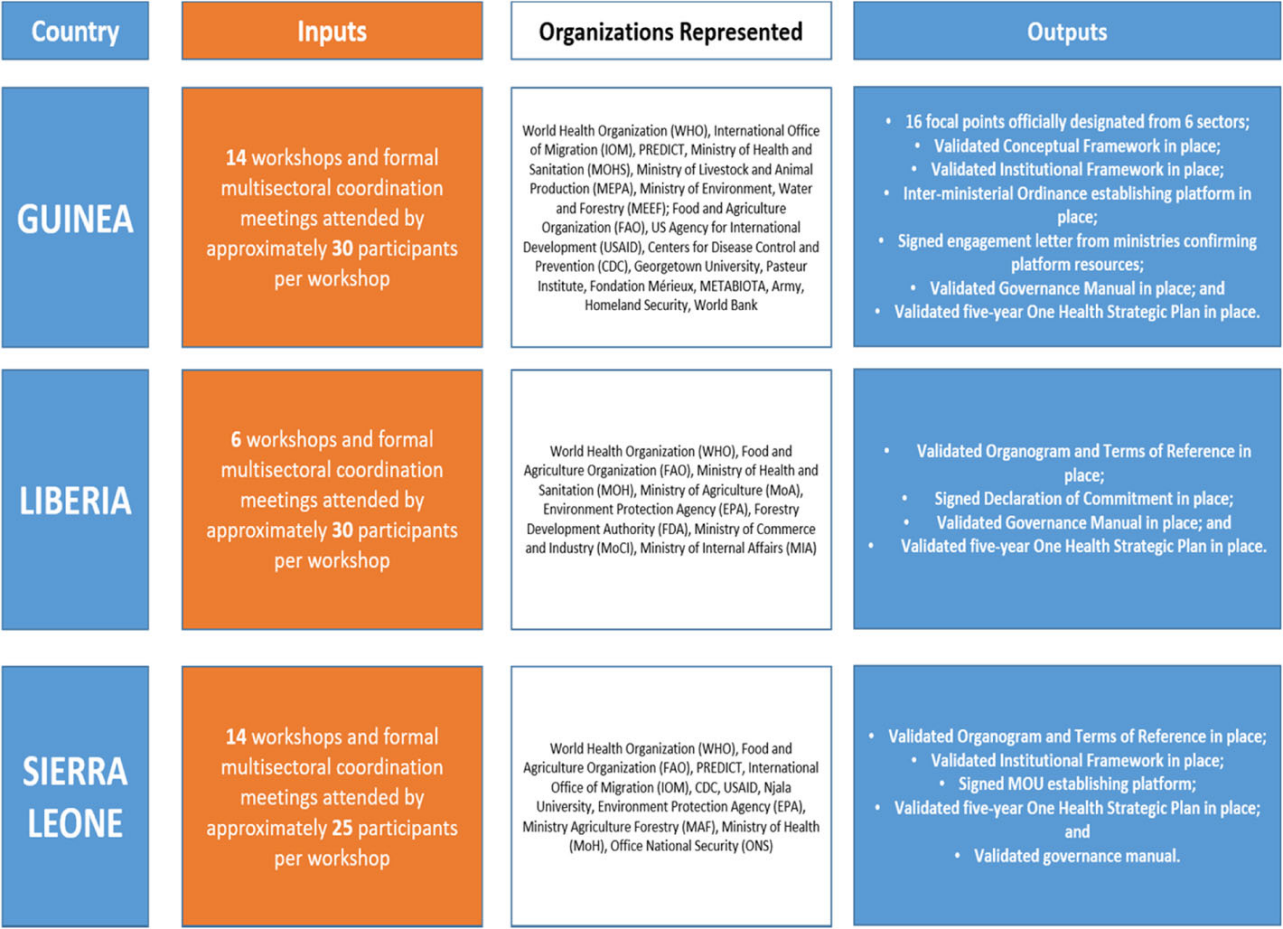
Summary of Key Inputs and Outputs of the National One Health Platforms in Guinea, Liberia, and Sierra Leone

Guided by robust governance manuals which provide detailed operational guidance to Platform leaders, Guinea, Liberia, and Sierra Leone have made important progress towards resource mobilization. For example, all three countries have secured support from the World Bank REDISSE project to strengthen their integrated surveillance systems, which provides a baseline from which governments will continue to build national capacity to prevent, detect, and respond to public health threats. To secure domestic financial allocations through annual ministry and agency budgets, the three West African nations have or are in the process of developing annual action plans identifying target activities from the five-year One Health Strategic Plan. Though progress with these annual One Health action plans varies among the countries, each has already achieved high level policy agreement on national ownership and continued investment in multisectoral activities. See Fig. 4 below:

### One health assessment for planning and performance (OH-APP) results to date

The latest OH-APP assessment results for Guinea, Liberia, and Sierra Leone are summarized in Table 5 below.

**Table 5:** One Health Assessment for Planning and Performance Results in Guinea, Liberia, and Sierra Leone

Indicator	Guinea (February 2018)	Liberia (October 2018)	Sierra Leone (May 2018)
*Capacity*			
Organizational Structure	3	3	2.5
Leadership	2	4	3
Multisectoral engagement	2.5	2.5	2
Communication and information exchange	2.3	1.6	2.3
Monitoring and evaluation	1	1	2
Government funding sources	1.5	2.5	1
Average – Capacity	2.1	2.4	2.1
*Performance*			
Engaging stakeholders	2.3	2.6	2
Coordinating and collaborating	2.3	3	1.5
Policy advocacy to overcome policy constraints	1.6	2.6	1
Joint planning among One Health stakeholders	1.8	2	1.9
Facilitating data analysis and use in decision-making	2	1	1
Mobilizing and optimizing resources	1	1	2
Average – Performance	1.8	2.0	1.6
Platform Level*	Beginning	Developing	Beginning

*The final scores for each capacity and performance indicator were established by consensus. Non-integer values represent partial status achievement for certain sub-indicators. For example, Guinea’s average capacity score of 2.1 indicates that it has achieved the minimum necessary capacity and initiated the government budget al*location process to begin “expanding,” but its performance score of 1.8 indicates that it has not yet demonstrated high performance. Together, these scores indicate that Guinea’s platform as a whole is still in the “Beginning” stage. The score is intended to motivate further action*

The three platforms have taken important steps to strengthen capacity and performance, but results indicate that they should continue to focus on monitoring and evaluation, resource mobilization, policy advocacy, and joint planning, among other actions, in order to become more functional.

## Challenges

Establishment and institutionalization of National One Health Platforms represented a departure from “business as usual” in addressing health threats in Guinea, Liberia, and Sierra Leone. Below are the major challenges encountered and an explanation of how they were addressed.

### Defining the platform and its structure

Broadly, a Platform serves as a multisectoral coordination mechanism (MCM) designed to address public health events/threats including emerging diseases, endemic diseases, AMR, disasters and environmental toxins among others. The decisions on who are the “key” platform stakeholders, and who should own and lead the Platform, were resolved by consensus in Guinea, Liberia, and Sierra Leone through stakeholder mapping followed by a long series of sensitization workshops and advocacy meetings with high-level national counterparts. The process was lengthy due to perceived challenges to individual sectoral leadership, competing priorities, and political transitions, among other roadblocks.

Often, government positions and functions are politically driven, and lack clarity or emphasis on accountability, efficiency, and effectiveness. Initially, each country anticipated following the usual hierarchical structure and retaining the independence of each individual sector, without considering the impact this would have on operational practicability, efficacy and efficiency in tackling public health events. Little was understood about the importance of new, cross-cutting structures such as the platform secretariat. During discussions defining the organizational structure of the Platform, national counterparts often attempted to suggest individuals for roles or responsibilities based on level of influence. Though no institution wanted to be left out of decision-making, few were accustomed to contributing to ongoing discussions moving forward a multisectoral agenda and did not know how to advocate for structural changes that would facilitate this type of collaboration.

These attitudes created a lot of confusion and frustration between the sectors, which was only resolved through a carefully executed series of sensitization sessions and other related multisectoral coordination meetings. During sessions facilitated by P&R staff and consultants, stakeholders worked backward from the management of a public health event to determine the key institutions and adequate functions which would have been necessary to properly manage the incident. Through continuous sensitization sessions and repeated discussion emphasizing the complementarity between government agencies and institutions, the importance of the structure and that of each actor to prevent, detect, and respond to public health events, tensions softened. A platform structure promoting institutional collaboration was ultimately established in Guinea, Liberia, and Sierra Leone.

### Achieving long-term multisectoral coordination through the platform

Multisectoral coordination is an ongoing challenge for all Platforms, given the need to balance individual ministry priorities with cross-cutting issues. Platforms must define methods for coordinating between different represented sectors but maintain leadership and ownership of existing activities and structures such as Emergency Operations Centers, which in many countries, such as Liberia, are managed by the public health sector.

The Prime Minister’s office or any other office at the highest policy level like the president’s office serves as the ideal coordinating body because of its primary mandate to coordinate all government sectors. However, there is no one size fit all and countries have the final decision on where they may anchor their platforms [20–23]. For example, Uganda agreed to establish a rotating coordination system, building on lessons from its success in controlling Trypanosomiasis [21]. In this scenario, equity in managing the Platform is achieved by giving each sector a designated period to coordinate the platform, which will empower each ministry and build leadership capacity. Kenya is another success story, where the platform has been anchored at the Ministry of Health for quite some time and has been functioning well [20, 22, 23].

An important lesson is that continuous strategic advocacy for coordination, joint planning and implementation is necessary when dealing with an environment where ministries are accustomed to jealously guarding their resources and viewing the gain of other structures as a loss to their own. This requires time, patience and always making sure that all key stakeholders are appropriately engaged in activity planning and implementation and feel heard when important decisions are made.

Highly motivated and respected national One Health “champions” are critical in these advocacy efforts. Champions are generally decision-makers or technical experts who have played a key role in raising awareness of the importance of multisectoral collaboration and push this topic as a national political priority.

### Integrating sectoral priorities into the platform

Though multisectoral in nature, the Platform is intended to complement individual sectoral ministries, who are represented on the Platform by their policy and decision makers. Representatives from each key One Health ministry form technical working groups which jointly plan and implement activities. Therefore, the Platform helps pool ministry-level knowledge and resources, facilitating discussions on how to leverage these resources for preparedness and response activities. It is well-positioned to convene discussions on joint resource mobilization and planning, ensuring that every sector benefit from new partnerships and other available shared resources.

While investing in multisectoral coordination, it is important for Platform leadership to continue making sectoral representatives feel empowered to contribute to the conversation and integrate knowledge, expertise, and concerns from individual sectors into Platform activities.

## Support from international partners

National governments assure long-term funding and technical support for the Platforms through annual budgetary allocations, which are essential for the resilience of national health systems to public health threats. International partners complement these efforts. The P&R project worked closely with international stakeholders such as the Food and Agriculture Organization (FAO), World Health Organization (WHO), and United States’ Centers for Disease Control and Prevention (CDC) in addition to other USAID/Emerging Pandemic Threats 2 partners. This strengthens complementarity among donor-funded initiatives, so that their support is in line with the 2017 commitments of African heads of states to accelerate implementation of the International Health Regulations (IHR 2005) and builds upon a strong foundation for future development [11].

A significant resource for One Health activities in West Africa is the World Bank REDISSE project, which provides support through the West African Health Organization (WAHO). Guinea, Liberia, and Sierra Leone have benefited from REDISSE funding since 2017 [14]. REDISSE has heavily participated in the process of establishing and maintaining the Platforms. Continued support from REDISSE offers a “bridge” for continued international support at this critical moment, allowing the One Health platforms enough time to more fully establish their presence. REDISSE’s M&E indicator related to financing the establishment and functionality of platforms illustrates its concrete commitment to ensuring their sustainability while local counterparts continue to advocate for national budget allocations [15].

## Conclusions and recommendations

There is documented evidence that “coordinated investigations between One Health sectors yield higher statistical power to elucidate public health relationships as compared to silo investigations and that the approach can result in improved resource efficiency” [20–25]. Greater preparedness and response can save lives, and prevent the economic catastrophes of uncontrolled health security events [22].

The Platforms in Guinea, Liberia, and Sierra Leone were established to address serious gaps in preparedness and response evidenced by the 2014–2016 Ebola crisis, and are anchored firmly within permanent national government structures. Stakeholders jointly determine, through consensus, the structure and composition of their platforms, formal legislation guaranteeing legal statuses, and develop critical tools such as National One Health Strategic Plans among others to guide multisectoral coordination over the next 5 yrs. The P&R project’s experience has demonstrated the importance of country ownership over the process for success: national stakeholders representing the human, animal, and environmental health sectors were successful in developing a Platform structure and prioritized action plans in accordance with national needs. For example, Sierra Leone has mobilized national and external support to revitalize its disease surveillance system, while at the same time it is a partner with other West African countries in the REDISSE project [26]. It is highly recommended that international and national stakeholders continue to support ongoing needs for resource mobilization, monitoring and evaluation, policy advocacy, and joint planning to further strengthen their National One Health Platforms and ensure that gains are sustained.

## Data limitations

The work described in this paper was conducted over a period of 3 yrs (June 2016–January 2019. Multisectoral coordination is a long-term process, and it must be acknowledged that there are limitations to the conclusions that can be drawn from this short experience. However, the authors wish to highlight the process and outputs so as to inspire other countries to initiate the establishment of National One Health Platforms, and attract continued support to sustain the current momentum in Guinea, Liberia, and Sierra Leone.

In a short period of time, the governments of Guinea, Liberia, and Sierra Leone succeeded in developing National One Health Strategic Plans. However, the P&R project ended prematurely before true implementation of prioritized activities began. This is an important area of follow-up, for both national and international stakeholders.

The absence of an outbreak in this short period also means that there is no true “case study” (outbreak response) to test improvements in national capacity to address a public health event in Guinea, Liberia, or Sierra Leone.

## Data Availability

Data and material is accessible at the USAID U.S. Agency for International Development’s Emerging Pandemic Threats 2 Program’s P&R project website http://preparednessandresponse.org and the Development Alternatives Inc. (DAI) archives. Authors may avail data and material on reasonable demand and with permission of DAI and USAID.

## Abbreviations

CDC: Centers for Disease Control and Prevention
ECOWAS: Economic Community of West African States
EPA: Environment Protection Agency
EPT-2: Emerging Pandemic Threats-2 program
FAO: Food and Agriculture Organization
FDA: Food and Drug Administration
GHSA: Global Health Security Agenda
IHR: International Health Regulations
IOM: International Office of Migration
JEE: Joint External Evaluation
MAF: Ministry of Agriculture Forestry
MEEF: Ministry of Environment, Water and Forestry
MEPA: Ministry of Livestock and Animal Production
MIA: Ministry of Internal Affairs
MoA: Ministry of Agriculture
MoC: Ministry of Commerce
MoH: Ministry of Health
MOHS: Ministry of Health and Sanitation
MoU: Memorandum of Understanding
NOHP: National One Health Platform
NOHSP: National One Health Strategic Plan
OH: One Health
OIE: Office International des Epizooties
ONS: Office of National Security
P&R: Preparedness and Response
REDISSE: The World Bank project on Disease surveillance in West Africa
ToR: Terms of Reference
USAID: United States Agency for International Development
WAHO: West African Health Organization
WHO: World Health Organization

## References

[1] Elmahdawy M, Elsisi GH, Carapinha J, Lamorde M, Habib A, Agyie-Baffour P (2017). Ebola virus epidemic in West Africa: Global Health economic challenges, lessons learned, and policy recommendations. Value in Health Regional Issues.

[2] World Bank Group. 2014-2015 West Africa Ebola crisis: impact update. World Bank Fiscal Report. 2016;4 Retrieved from http://pubdocs.worldbank.org/en/297531463677588074/Ebola-Economic-Impact-and-Lessons-Paper-short-version.pdf.

[3] The Lancet (2014). Ebola in west Africa: gaining community trust and confidence. The Lancet.

[4] Heymann DL, Chen L, Takemi K, Fidler DP, Tappero JW, Thomas MJ (2015). Global health security: the wider lessons from the west African Ebola virus disease epidemic. Lancet.

[5] The Global Health Security Agenda (GHSA). https://www.ghsagenda.org/

[6] Kieny MP, Evans DB, Schmets G, Kadandale S (2014). Health-system resilience: reflections on the Ebola crisis in western Africa. Bull World Health Organ.

[7] Report on the One Health Technical and Ministerial Meeting to Address Zoonotic Diseases and Related Public Health Threats. https://reliefweb.int/report/world/report-one-health-technical-and-ministerial-meeting-address-zoonotic-diseases-and

[8] Report on One Health technical and ministerial meeting to address zoonotic diseases and related public health threats (November 2016). Website lats accessed 30^th^ July 2019. https://www.afro.who.int/sites/default/files/2018-02/Report%20of%20the%20One%20Health%20Technical%20and%20Ministerial%20Meeting%20%2D%2D%20Dakar_.pdf

[9] Organisation Oest Africaine de la Sante (June 2017). ECOWAS Ministers in charge of Health, Agriculture and Environment meet to adopt the 'One Health' Policy Coordination Platform. Website last accessed 30^th^ July 2019. http://w3.wahooas.org/web-ooas/fr/actualites/ecowas-ministers-charge-health-agriculture-and-environment-meet-adopt-one-health-policy

[10] FAO and ECOWAS support One Health approach for health security in West Africa, Dakar, November 2016. Website last accessed 30^th^ July 2019. http://www.fao.org/africa/news/detail-news/en/c/452342/

[11] Nkengasong J, Djoudalbaye B, Maiyegun O (2017). A new public health order for Africa’s health security. Lancet Glob Health.

[12] World Health Organization. (2005). International Health Regulations. Who, 82. 10.1177/146642407109100301

[13] The OIE *Terrestrial Animal Health Code* (the *Terrestrial Code*). http://www.oie.int/international-standard-setting/terrestrial-code/

[14] World Bank (2016). Africa - regional disease surveillance systems enhancement (REDISSE) project (English).

[15] World Bank (2016). Website accessed 20^th^ August 2019. http://documents.worldbank.org/curated/en/965001467305866621/pdf/PAD1752-PAD-P154807-OUO-9-IDA-R2016-0154-1-Box396265B.pdf

[16] Preparedness & Response project. Website last accessed 30^th^ July 2019. https://drive.google.com/open?id=1XMLYGR0nK8djaLj24Mnsv2SY-rj5KCm9

[17] Preparedness and Response project. Website last accessed 30^th^ July 2019. https://www.onehealthapp.org/resources and https://www.onehealthapp.org/about

[18] CDC: Zoonotic Disease Prioritization, Website last accessed 2^nd^ August 2019. https://www.cdc.gov/onehealth/global-activities/prioritization-workshop.html

[19] A Tripartite Guide to Addressing Zoonotic Diseases in Countries - 0IE. http://www.oie.int/fileadmin/Home/eng/Media_Center/docs/EN_TripartiteZoonosesGuide_webversion.pdf

[20] Okello AL, Bardosh K, Smith J, Welburn SC (2014). One health: past successes and future challenges in three African contexts. PLoS Negl Trop Dis.

[21] Coordinating Office for Control of Trypanosomiasis in Uganda (COCTU). http://www.coctu.go.ug/

[22] Munyua PM, Njenga MK, Osoro EM, Onyango CO, Bitek AO, Mwatondo A, et al. Successes and challenges of the one health approach in Kenya over the last decade. BMC public health 2019, 19 (Suppl3): 465. 2019. 10.1186/s12889-019-6772-7. 10.1186/s12889-019-6772-7 PMC6696663 32326940

[23] Mwatondo A, Munyua P, Gura Z, Muturi M, Osoro E, Obonyo M (2017). Catalysts for implementation of One Health in Kenya. Pan African Medical Journal.

[24] World Bank (2012). PEOPLE, PATHOGENS AND OUR PLANET volume 2 - the economics of one health. The World Bank.

[25] Rostal MK, Ross N, Machalaba C, Cordel C, Paweska JT, Karesh WB (2018). Benefits of a one health approach: an example using Rift Valley fever. One Health.

[26] Njuguna C, Jambai A, Chimbaru A, Nordstrom A, Conteh R, Latt A, O-tipo S, et al ( 2019). Revitalization of integrated disease surveillance and response in Sierra Leone post Ebola virus disease outbreak. BMC Public Health (2019) 19:364. 10.1186/s12889-019-6636-1https://bmcpublichealth.biomedcentral.com/articles/10.1186/s12889-019-6636-1 10.1186/s12889-019-6636-1 PMC6444503 30940125

